# Effect of steroids and relevant cytokine analysis in acute tubulointerstitial nephritis

**DOI:** 10.1186/s12882-019-1277-2

**Published:** 2019-03-12

**Authors:** Donghwan Yun, Myoung-jin Jang, Jung Nam An, Jung Pyo Lee, Dong Ki Kim, Ho Jun Chin, Yon Su Kim, Dong-Sup Lee, Seung Seok Han

**Affiliations:** 1 Department of Internal Medicine, Seoul National University College of Medicine, Seoul, South Korea; 2 Medical Research Collaborating Center, Seoul National University Hospital, Seoul, South Korea; 3 Department of Internal Medicine, Seoul National University Boramae Medical Center, Seoul, South Korea; 4 Department of Internal Medicine, Seoul National University Bundang Hospital, Seongnam-si, Gyeonggi-do South Korea; 5 Department of Biomedical Sciences, Seoul National University College of Medicine, Seoul, South Korea

**Keywords:** Acute tubulointerstitial nephritis, Cytokine, End-stage renal disease, Mortality, Steroid

## Abstract

**Background:**

Acute tubulointerstitial nephritis (ATIN) is an important cause of acute kidney injury and often a potentially reversible disease. However, the role of steroids in ATIN remains controversial and the underlying mechanisms remain unresolved.

**Methods:**

A total of 113 adult patients with biopsy-proven ATIN were recruited from three tertiary referral centers. Of 102 patients with idiopathic or drug-induced ATIN, outcomes such as renal recovery, end-stage renal disease, and all-cause mortality were compared between the steroid-treated and non-treated groups. Plasma and urine inflammatory cytokine levels at the time of biopsy were analyzed in patients (*n* = 33) using a bead-based multiplex assay and compared with those of healthy individuals (*n* = 40).

**Results:**

Steroids were used in 92 (81.4%) of the total patients and in 82 (80.3%) patients with idiopathic or drug-induced ATIN. The rate of renal recovery and the risks of end-stage renal disease and mortality were not different between the steroid-treated and non-treated groups. Despite using a propensity score matching method (*n* = 20 in each group), none of the outcomes were different between the two groups. Several cytokines, such as monocyte chemotactic protein-1, interferon-α, and interleukin-6 and interleukin-8 levels, were markedly elevated in plasma and urine of patients compared with those in healthy individuals. However, cytokines related to Th2 response, such as IL-10, IL-33, were not different between the two groups.

**Conclusions:**

Steroid use does not affect the overall outcome of ATIN. Based on the fact that targeting therapy should be investigated to improve outcomes, the present cytokine results will be helpful for developing a novel therapy for ATIN.

**Electronic supplementary material:**

The online version of this article (10.1186/s12882-019-1277-2) contains supplementary material, which is available to authorized users.

## Background

Acute tubulointerstitial nephritis (ATIN) is an important cause of acute kidney injury, and it causes inflammation and dysfunction mainly in the tubules, without affecting glomeruli and vasculature. ATIN accounts for 0.5**–**2.6% of all kidney biopsies and 5**–**27% of unexplained acute kidney injury [1–4]. The etiology of ATIN includes drug-induced, autoimmune disease, infection, malignancy, and others [5]. ATIN was originally thought to be mainly caused by infection, but a drug-induced cause has become the main etiology in recent years [4, 6–8].

With regard to the pathogenesis of ATIN, a hypersensitivity reaction to kidney antigens is thought to play a major role. ATIN occurs only in a small percentage of individuals and in a dose-independent manner, and sometimes exhibits extra-renal manifestations related to hypersensitivity [9, 10]. However, the underlying mechanism of ATIN is not fully understood. In previous retrospective reviews, steroids were mostly used or recommended to be used for ATIN [11–17]. Additionally, oral prednisolone at 1 mg/kg/day for ATIN was usually used with or without pulse therapy of methylprednisolone over the next 3**–**12 weeks [4]. However, there is no clear consensus on the effectiveness of steroids because there have been no prospective, randomized, controlled trials of steroid use in relation to the clinical course of ATIN to date.

Cohort studies on patients with ATIN and possible effects of steroids have been conducted [11–16]. However, further studies on patients with ATIN and various characteristics are required to determine the significance of steroid capacity. Research on the underlying mechanism may also provide information on this issue. The present cohort study aimed to examine the effect of steroids on renal outcomes, including recovery of ATIN, and the risks of end-stage renal disease (ESRD) and mortality. Subsequent analyses of cytokine and chemokine, which are possibly related to tubular inflammation, were conducted to provide a clue of the pathophysiology of ATIN.

## Methods

### Patients and data collection

The study design was approved by the institutional review boards of all involved centers (nos. H-1801-043-914, B-1801/447–409, and 20,180,124/30–2018-3/023) and complied with the Declaration of Helsinki. Informed consents were waived under this approval. A total of 7196 patients had a kidney biopsy performed at three tertiary referral hospitals (Seoul National University Hospital, Boramae Medical Center, and Seoul National University Bundang Hospital) over 18 years (from January 2000 to December 2017). Among them, 270 patients were diagnosed with ATIN. Patients were excluded from the analysis if they also had glomerulonephritis (*n* = 122), pyelonephritis (*n* = 3), or other pathologies, including thrombotic microangiopathy (*n* = 6) and atheroembolism (*n* = 1), they were younger than 18 years (*n* = 5), and they had an insufficient follow**-**up period (< 3 months) (*n* = 11). We also excluded patients without azotemia at the time of biopsy (*n* = 9: 5 were renal tubular acidosis, 2 were drug-induced ATIN, 1 was renal tuberculosis, and 1 was related with Sjögren syndrome) because the effect of steroids on outcomes of ATIN could not be examined in these patients. Finally, the analyzed cohort comprised 113 patients.

All of the patients’ clinical data such as age, sex, body mass index, comorbidities, including diabetes mellitus, hypertension, and chronic kidney disease, renal outcomes, and steroid treatment for ATIN, were collected. Baseline blood laboratory data, such as serum creatinine, hemoglobin, cholesterol, uric acid, and albumin levels, were measured. The dipstick test was used to evaluate proteinuria, which was semi-quantitatively scored. Microscopic evaluation of urine was performed to define hematuria and pyuria. The random urine protein to creatinine ratio was measured at the time of biopsy, but this variable was not available in five patients. The etiology of ATIN was classified into idiopathic if there were no specific factors inducing kidney dysfunction. Two nephropathologists reviewed all of the slides and semi-quantitative graded scores for tubular atrophy and interstitial fibrosis, and leukocyte infiltration, according to the Banff working classification [18].

Renal recovery, which was primary outcome, was defined as a decrease in serum creatinine levels to ≤1 .3mg/dL or ≥ 50% from its peak value. Renal recovery was evaluated at multiple time points, such as at 6 months after kidney biopsy and at the last follow-up. The outcome of ESRD was defined as initiation of renal replacement therapy or kidney transplantation due to failed kidney function. The events of ESRD and all-cause mortality were obtained from the Kidney Renal Registry and the National Database of Statistics Korea, respectively.

### Cytokine analysis

Thirty three patients’ plasma and urine were acquired at the time of kidney biopsy and before any treatment was provided. Forty healthy individuals’ plasma and urine were also recruited at the time of a health checkup for comparison with the patients. We performed a bead-based multiplex assay according to the manufacturer’s instruction (LEGENDplex™; BioLegend, San Diego, CA, USA). This assay included 13 cytokines and chemokines which were known to play a crucial role in renal ischemia-reperfusion injury [19–22] and were possibly related to tubular inflammation pathways [23], such as interleukin (IL)-1β, interferon (IFN)-α2, IFN-γ, tumor necrosis factor-α, monocyte chemo-attractant protein-1, IL-6, IL-8, IL-10, IL-12p70, IL-17A, IL-18, IL-23, and IL-33. Cytokine- and chemokine-bound beads were read on a flow cytometer (BD LSRFortessa™; BD Biosciences, San Jose, CA, USA). Data were analyzed using the analysis program recommended by the manufacturer’s instructions (LEGENDplex™, Data Analysis v8.0; VigeneTech, Carlisle, MA, USA).

### Statistical analysis

All statistical analyses were performed using SPSS version 23.0 (IBM Corp., Armonk, NY, USA) and R software (version 3.4.1; The Comprehensive R Archive Network: http://cran.r-project.org). Continuous variables are expressed as the mean value and standard deviation if they had a normal distribution and as the median value and interquartile range if they were not normally distributed. A normality test was performed using the Kolomogrov–Sirnov test. Categorical variables are expressed as proportions. The chi-square test was used for comparison of categorical variables (Fisher’s exact test if not applicable) and the Student’s t-test was used for continuous variables. The Mann–Whitney test was used if variables were not normally distributed. Analysis of variance analysis was used when normality was satisfied for comparison between group, and the Kruskal–Wallis test was used if normality was not satisfied. Logistic and Cox proportional hazard ratio models were conducted to calculate odds ratios (ORs) and hazard ratios (HRs) of the outcome risk, respectively. All of the covariates shown in Table 1, except for the random urine protein to creatinine ratio (missing in five cases), were adjusted in the multivariate models. To assess the effect of steroids in ATIN, we pooled the data of three previous retrospective cohort studies, and compared the ESRD progression rate between the steroid-treated and non-treated groups. Pooled estimates of the relative risks and 95% confidence intervals (95% CIs) were obtained using the DerSimonian and Laird random effects model. Heterogeneity was assessed using the Cochran Q statistic and I^2^. All *P* values were set to two-sided, and values ≤0.05 were defined as significant.

**Table 1:** Baseline characteristics of study subjects according to the etiology

Variable	All patients (*n* = 113)	Idiopathic (*n* = 77)	Drug induced (*n* = 25)	Autoimmune (*n* = 5)	Others^a^ (*n* = 6)	*P*-value^b^
Age (year)	58.3 ± 15.2	57.2 ± 15.4	62.7 ± 12.8	58.0 ± 22.6	54.7 ± 15.4	0.428
Female (%)	40.7	45.5	28.0	20.0	50.0	0.338
Body mass index (kg/m^2^)	23.3 ± 3.6	23.5 ± 3.4	23.2 ± 4.1	22.9 ± 4.0	20.5 ± 2.5	0.263
Comorbidities (%)						
Diabetes mellitus	30.1	35.1	20.0	0	33.3	0.255
Hypertension	40.7	41.6	44.0	40.0	16.7	0.687
Chronic kidney disease	11.5	9.1	20.0	0	16.7	0.330
Blood findings						
Peak sCr (mg/dL)	4.50 (3.02**–**7.23)	4.35 (3.02**–**6.40)	5.42 (3.70**–**9.70)	2.61 (2.50**–**7.02)	5.36 (3.94**–**7.85)	0.362
sCr at biopsy (mg/dL)	3.77 (2.51**–**5.90)	3.50 (2.67**–**5.58)	3.91 (3.02**–**8.80)	2.50 (2.04**–**5.93)	4.54 (2.48**–**7.68)	0.528
Hemoglobin (g/dL)	10.1 ± 1.7	10.0 ± 1.6	10.4 ± 1.8	10.2 ± 1.9	10.1 ± 1.6	0.785
Cholesterol (mg/dL)	139.0 (119.0**–**165.0)	139.0 (121.0**–**167.0)	143.0 (116.0**–**166.0)	127.0 (111.0**–**146.0)	127.0 (101.0**–**149.0)	0.338
Uric acid (mg/dL)	6.1 (4.6**–**8)	5.9 (4.2**–**8)	6.3 (5.1**–**8)	5.3 (3.8**–**7.4)	7.6 (5.1**–**8.7)	0.665
Albumin (g/dL)	3.5 (3**–**3.9)	3.5 (3.1**–**3.9)	3.3 (2.9**–**3.9)	2.4 (2.3**–**3.7)	3.7 (3.5**–**3.9)	0.279
Proteinuria (%)						0.886
- or trace	27.4	31.2	20.0	20.0	16.7	
1+	36.3	32.5	36.0	60.0	66.7	
2+	24.8	24.7	28.0	20.0	16.7	
≥ 3+	11.5	11.7	16.0	0	0	
Hematuria (%)	40.7	35.1	60.0	40.0	33.3	0.175
Pyuria (%)	58.4	57.1	60.0	60.0	66.7	1.000
uPCR (g/g)	1.3 (0.7**–**2.5)	1.3 (0.8**–**2.5)	1.3 (0.7**–**4.7)	1.1 (1.0**–**1.5)	1.7 (1.2**–**1.9)	0.865
Dialysis at biopsy (%)	30.1	24.7	44.0	40.0	33.3	0.251
Steroid use (%)	81.4	79.2	84.0	100	83.3	0.892
TA/IF (%)						0.378
None	15.9	10.4	24.0	20.0	50.0	
Milde	33.6	33.8	36.0	40.0	16.7	
Moderate	39.8	44.2	28.0	40.0	33.3	
Severe	10.6	11.7	12.0	0	0	
Leukocyte infiltration (%)						0.906
Mild	17.7	15.6	24.0	20.0	16.7	
Moderate	38.1	41.6	28.0	40.0	33.3	
Severe	44.2	42.9	48.0	40.0	50.0	
Follow-up duration (months)	33 (19**–**55)	37 (19**–**54)	24 (19**–**60)	48 (30**–**56)	16 (8**–**24)	0.316

Data are *n* (%) or mean (±standard deviation) or median (interquartile range)

*sCr* serum creatinine, *uPCR* random urine protein to creatinine ratio, *TA/IF* tubular atrophy and interstitial fibrosis

^a^Others included 5 cases of malignancy; and 1 case of infection

^b^*P*-value was obtained from Kruskal-Wallis test or ANOVA test between groups

## Results

### Baseline characteristics

Baseline characteristics of the patients are shown in Table 1. The etiology of ATIN was idiopathic in 77 (68.1%) patients, drug-induced in 25 (22.1%), autoimmune in five (4.4%), malignancy in five (4.4%), and infection in one (0.8%). In the case of drug-induced etiology, herbal medication was the most common cause (*n* = 8, 32%), followed by nonsteroidal anti-inflammatory drugs or salicylate (*n* = 6, 24%), antibiotics (*n* = 6, 24%), proton pump inhibitors (*n* = 2, 8%), and others (2 cases of oxaliplatin, 1 case of tranexamic acid). In the case of autoimmune ATIN, there were three cases of anti-neutrophil cytoplasmic antibody-associated vasculitis, one case of tubulointerstitial nephritis and uveitis, and one case of Sjögren syndrome. All cases of malignancy (*n* = 5) were multiple myeloma, and infectious-ATIN was associated with Hantaan virus.

The mean age of the patients was 58 (±15) years and 60% of them were men. At the time of biopsy, the median value of serum creatinine was 3.77 mg/dL and the peak level was 4.50 mg/dL. Dialysis at the time of biopsy was performed in 30.1% of patients, and steroids were used for 81.4% of patients. Patients with autoimmune disease or malignancy were treated with steroids, but the patient with infectious ATIN was not treated. The patients were followed up for a median of 33 months (interquartile range, 19–55 months; maximum of 12 years).

### Overall renal outcomes

Table 2 shows the overall renal outcomes according to the etiology of ATIN. The rate of renal recovery was 54.9% at 6 months after biopsy and 73.5% during the entire follow-up period. The rate of ESRD progression was 25.7% and the all-cause mortality rate was 17.7% in all patients. Of five patients with malignancy-associated ATIN who were included in the others category, four progressed to ESRD and died. This was the reason for differences in the rates of renal recovery and mortality among the groups.

**Table 2:** Renal outcome classified by the etiology

Outcome	All patients (*n* = 113)	Idiopathic (*n* = 77)	Drug induced (*n* = 25)	Autoimmune (*n* = 5)	Others (*n* = 6)	*P*-value^a^
Renal recovery at 6 months (%)	54.9	53.2	68.0	40.0	33.3	0.333
Renal recovery at last follow-up (%)	73.5	71.4	88.0	80.0	33.3	0.043
End-stage renal disease (%)	25.7	24.7	20.0	20.0	66.7	0.088
All-cause mortality (%)	17.7	15.6	16.0	0.0	66.7	0.029

^a^*P*-value was measured using the chi-square test between all groups

### Effect of steroids on renal outcome

Steroid use in cases of autoimmune diseases and multiple myeloma was essential for treating the diseases, and steroid use in infectious disease was usually not recommended. Therefore, patients with autoimmune- and malignancy-associated, and infectious ATIN were excluded from the analyses to determine the effect of steroid use. To evaluate the effect of steroid use on outcomes, 102 patients with idiopathic and drug-induced ATIN were analyzed after stratification by the use of steroids. Steroids were used in 80.3% of patients, and the median value of initial steroid dose was 30 mg/day (30–60 mg/day as an equivalent dosage for prednisolone) and the median duration of use was 14 weeks (9–30 weeks). The steroid-treated group was having lower body mass index, less chronic kidney disease, lower uric acid level, and lower score of tubular atrophy and interstitial fibrosis than did the non-treated group (Table 3).

**Table 3:** Baseline characteristics of idiopathic or drug-induced acute tubulointerstitial nephritis

Variable	All subjects (*n* = 102)	Steroid-treated group (*n* = 82)	Non-treated group (*n* = 20)	*P*-value^a^
Age (year)	58.6 ± 14.9	58.1 ± 15.4	60.6 ± 13.0	0.501
Female (%)	41.2	39.0	50.0	0.371
Body mass index (kg/m^2^)	23.4 ± 3.5	22.9 ± 3.4	25.4 ± 3.3	0.005
Comorbidities (%)				
Diabetes mellitus	31.4	29.3	40.0	0.354
Hypertension	42.2	37.8	60.0	0.072
Chronic kidney disease	11.8	7.3	30.0	0.012
Blood findings				
Peak sCr (mg/dL)	4.57 (3.04**–**7.23)	4.67 (3.00**–**7.46)	4.43 (4.06**–**6.05)	0.879
sCr at biopsy (mg/dL)	3.73 (2.70**–**5.80)	3.89 (2.51**–**6.10)	3.40 (3.09**–**4.05)	0.458
Hemoglobin (g/dL)	10.0 ± 1.6	10.5 ± 1.7	9.9 ± 1.6	0.201
Cholesterol (mg/dL)	140.0 (121.0**–**167.0)	140.0 (121.0**–**166.0)	144.0 (122.0**–**186.0)	0.820
Uric acid (mg/dL)	6.1 (4.6**–**8.0)	5.9 (4.1**–**7.5)	7.5 (6.2**–**9.9)	0.005
Albumin (g/dL)	3.5 (3.1**–**3.9)	3.5 (3.1**–**3.9)	3.2 (2.9**–**4.0)	0.280
Proteinuria (%)				0.116
- or trace	28.4	24.4	45.0	
1+	33.3	36.6	20.0	
2+	25.5	28.0	15.0	
≥ 3+	12.7	11.0	20.0	
Hematuria (%)	41.2	37.8	55.0	0.161
Pyuria (%)	57.8	56.1	65.0	0.470
uPCR (g/g)	1.2 (0.7**–**2.5)	1.3 (0.7**–**2.4)	0.9 (0.7**–**4.2)	0.676
Dialysis at biopsy (%)	29.4	30.5	25.0	0.629
TA/IF (%)				0.024
None	13.7	12.2	20.0	
Milde	34.3	37.8	20.0	
Moderate	40.2	42.7	30.0	
Severe	11.8	7.3	30.0	
Leukocyte infiltration (%)				0.514
Mild	17.6	15.9	25.0	
Moderate	38.2	37.8	40.0	
Severe	44.1	46.3	35.0	
Follow-up duration (months)	34 (19**–**55)	34 (18**–**54)	27 (21**–**69)	0.508

^a^*P*-value was obtained from the Student’s t-test or the Mann-Whitney test

**Fig. 1:**
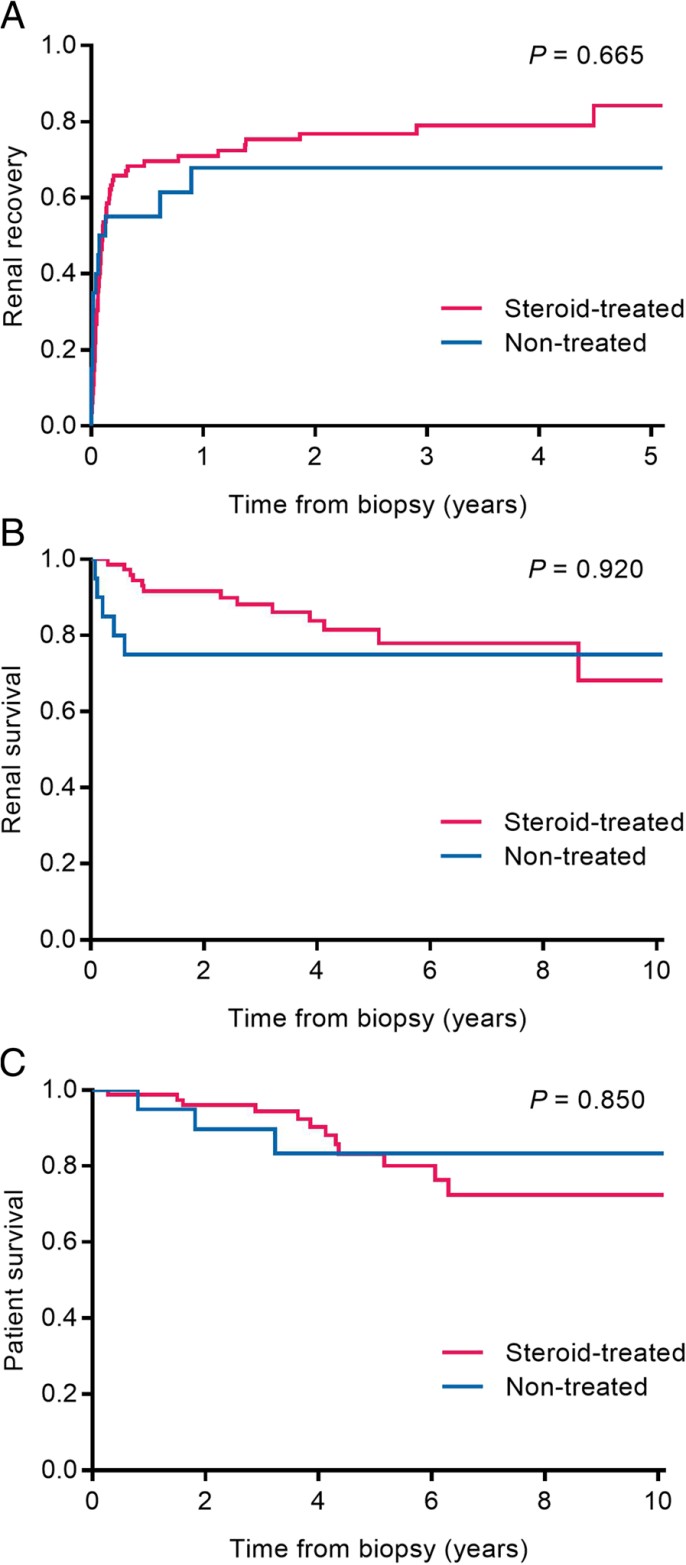
Meta-analysis of end-stage renal disease in the steroid-treated group compared with the non-treated group

Figure 1 shows the Kaplan–Meier curves of the rate of renal recovery and risks of ESRD and mortality depending on the use of steroids. None of the renal outcomes, including renal recovery and the risks of ESRD and all-cause mortality, were different between the steroid-treated and non-treated groups. Despite adjustment of multiple variables, the steroid-treated group did not have favorable outcomes compared with the non-treated group (Table 4). To balance all of the observed baseline variables between groups, we further used 1:1 propensity score matching. Propensity scores were estimated by logistic regression, with several variables, including age, sex, body mass index, diabetes mellitus, hypertension, chronic kidney disease, serum creatinine levels at biopsy, uric acid levels, albumin levels, proteinuria, hematuria, tubular atrophy and interstitial fibrosis, leukocyte infiltration, dialysis at biopsy, and serum creatinine levels at the time of kidney biopsy. The steroid-treated group had similar rates of renal recovery at 6 months (58.5% vs. 50.0%; OR, 1.05; 95% CIs 0.258–4.269; *P* = 0.947), renal recovery at the last follow-up (78.0% vs. 65.0%; HR 0.82; 95% CIs 0.401–1.674; *P* = 0.583), ESRD (23.2% vs. 25.0%; HR 0.76; 95% CIs 0.188–3.032; *P* = 0.693), and all-cause mortality (14.6% vs. 20.0%; HR 0.33; 95% CIs 0.049–2.174; *P* = 0.248) compared with the non-treated group (Table 4). In the regression models, diabetes mellitus, but not the other variables, was associated with the renal recovery rate (Additional file 1: Table S1).

**Table 4:** Renal outcome of unclear or drug-induced acute tubulointerstitial nephritis classified by steroid use

Outcome	All subjects (*n* = 102)	Non-treated group (*n* = 20)	Steroid-treated group (*n* = 82)	*P*-value^a^	Matched steroid-treated group (*n* = 20)	*P*-value
Renal recovery at 6 months (%)	56.9	50.0	58.5	0.947	50.0	1.000
Renal recovery at last follow-up (%)	75.5	65.0	78.0	0.583	75.0	0.490
End-stage renal disease (%)	23.5	25.0	23.2	0.693	35.0	0.490
All-cause mortality (%)	15.7	20.0	14.6	0.248	10.0	0.661

^a^Adjusted for age, sex, body mass index, diabetes mellitus, hypertension, chronic kidney disease, serum creatinine at kidney biopsy, uric acid, albumin, proteinuria, hematuria, tubular atrophy and interstitial fibrosis, leukocytes infiltration, and dialysis at biopsy

Subgroup analysis was performed in the steroid-treated group to determine whether factors related to steroid use, such as pulse therapy of methylprednisolone, initial steroid dose, and delay of starting steroids affected renal outcomes. After adjustment of multiple variables, none of variables were associated with renal recovery at 6 months, including methylprednisolone pulse therapy (OR 1.02 vs. none; 95% CIs 0.987–2.210; *P* = 0.276), initial steroid dose (OR 0.97 per 1 mg of prednisolone dose; 95% CIs 0.894–1.044, *P* = 0.381), and a delayed start of steroids (OR 1.00 per 1 day of delay; 95% CIs 0.964–1.041; *P* = 0.924).

### Pooled analysis

**Fig. 2:**
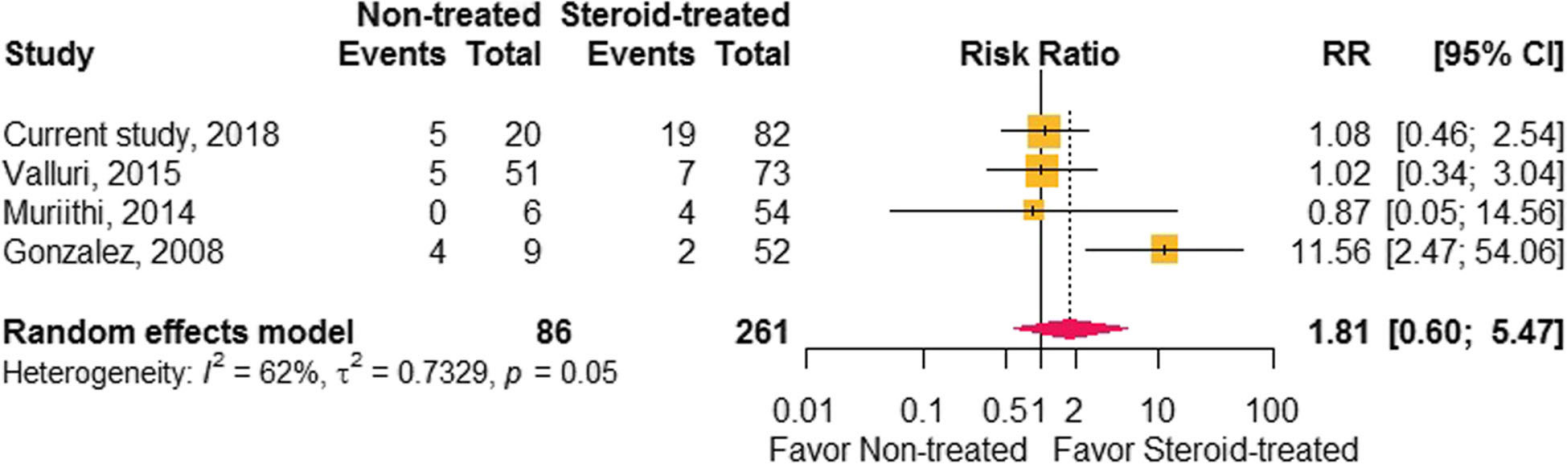
Kaplan Meier curves of renal recovery (**a**), end-stage renal disease (**b**), and patients’ survival (**c**) between the steroid-treated and non-treated groups. Differences were measured using the log rank test

We performed pooled analysis on the effects of steroids on the outcome of ESRD in drug-induced ATIN from three studies [12, 14, 15], as well as the present study (*n* = 347). We used the DerSimonian and Laird random effects model because of heterogeneity between studies. There was no significant difference in progression of ESRD between the steroid-treated and non-treated groups (relative risk, 1.81; 95% CIs 0.599–5.474; *P* = 0.292) (Fig. 2).

### Cytokine and chemokine analysis

**Fig. 3:**
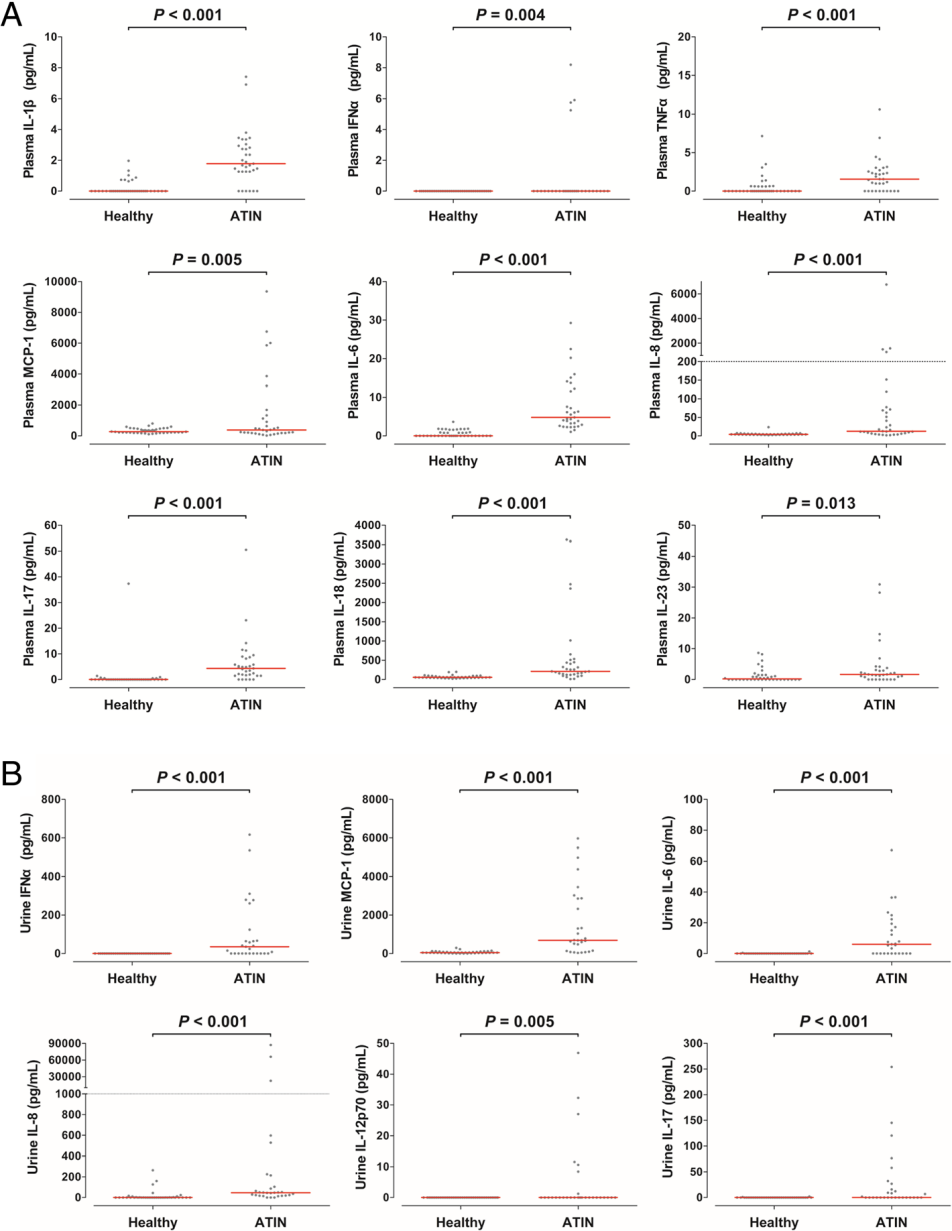
Cytokine and chemokine analysis in plasma (**a**) and urine (**b**) at the time of kidney biopsy in patients compared with samples of healthy individuals

The above results showed that steroid therapy did not affect the overall outcomes of ATIN. Accordingly, we need to adopt other treatment options to improve patient’s outcomes. Nevertheless, understanding of the pathophysiology of ATIN is not clear. Therefore, we attempted to determine which inflammatory cytokines or chemokines are expressed in patients with ATIN. Plasma levels of IL-1β, IFN-α2, tumor necrosis factor-α, monocyte chemo-attractant protein-1, IL-6, IL-8, IL-17A, IL-18, and IL-23 were higher in patients with ATIN than in healthy individuals (Fig. 3a). Urine levels of IFN-α2, monocyte chemo-attractant protein-1, IL-6, IL-8, IL-12p70, and IL-17A were higher in patients with ATIN than in healthy individuals (Fig. 3b). The other cytokines including IL-10, IFN-γ, IL-23, IL-33 were not different between patients and healthy individuals.

## Discussion

ATIN is an important cause of tubular injury, but its pathophysiology and treatment are not well understood. The present study evaluated the effect of steroids in patients with idiopathic and drug-induced ATIN. However, our results did not support an effect of steroids on renal outcomes and patients’ survival. For the first time, we performed cytokine and chemokine analyses in plasma and urine before steroid treatment. We found that certain cytokines and chemokines were elevated in patients with ATIN.

The present study showed that renal outcomes of ATIN were significantly different depending on the etiology. Drug-induced ATIN showed the most favorable outcome (88.0% of renal recovery during the study period), and malignancy-associated ATIN was the worst (20%). The etiology should be considered to evaluate the effects of steroid. Therefore, drug-induced and idiopathic ATIN were included in analysis of the effect of steroids in our study. In these groups, steroid use did not appear to play a significant role in renal recovery. This trend remained consistent, even after matching was performed. Subgroup analysis of the steroid-treated group also showed that there was no significant difference in renal recovery according to pattern of steroid use, such as methylprednisolone pulse therapy, steroid dose, and a delayed start of steroids.

In drug-induced ATIN, steroids are recommended after culprit drug withdrawal [8]. Although inconsistent results have been shown in previous papers on the steroid effect of ATIN [4], steroids are generally used for treating ATIN. Previous retrospective studies related to the effect of steroids on ATIN that compared a steroid group and a non-steroid group are shown in Table 5 [11–16]. Most patients with ATIN in these studies were treated with steroids (59**–**87%), and different criteria were applied to evaluate renal recovery in each article. Three of these studies [12, 13, 16] showed that steroids were effective in renal outcome, while the other three [11, 14, 15] did not. Among the studies that analyzed the effect of steroids on drug-induced ATIN, only one study [12] showed a positive result of steroid use. These previous studies and the current study do not provide clear evidence to support steroid use.

**Table 5:** Summary of previous published articles regarding steroid effect in acute tubulointerstitial nephritis

Study	Case no.	Etiology	Steroid use (%)	Outcome analysis	Results
Prendecki, 2017	187	All etiologies^a^	84	Median eGFR comparison at 1, 3, 6, 12, 24 months, and last follow-up	The steroid-treated group had a significantly higher eGFR at 6, 12, 24 months and at last follow-up
Valluri, 2015	124	Drug induced	59	Median sCr comparison and renal recovery within 1 year (complete: return to baseline sCr)	No difference in sCr at time points of 1, 6, and 12 months and no difference in renal recovery between the two groups (48% vs. 41%)
Muriithi, 2014	95	Drug induced	87	Renal recovery at 6 months (complete: within 25% of its baseline or < 1 .4mg/dL if baseline was not available, partial: ≥50% decrease of peak sCr)	Treatment with steroids did not affect renal recovery status at 6 months (complete: partial: none = 49%: 39%: 12% vs. 17%: 67%: 16%; *P* = 0.3)
Raza, 2012	49	All etiologies^b^	76	Fold improvement in eGFR at last follow-up	Greater improvement in eGFR in patients with steroids (3.4 vs. 2.1; *P* < 0.05)
Gonzalez, 2008	61	Drug induced	85	Median sCr comparison and renal recovery (> 50% decrease of peak sCr) based on last follow-up	Significantly lower sCr in steroid group (2.1 vs. 3.7, *P* < 0.05), No significant change in renal recovery between two groups (54% vs. 33%, *P* = ns)
Clarkson, 2004	42	All etiologies^c^	62	Median sCr comparison at 1, 6, and 12 months	No difference in sCr at time points of 1, 6, and 12 months between the two groups
Yun, 2018	102	Idiopathic & drug induced	80	Renal recovery at 6 months (≥50% decrease of peak sCr or < 1 .3mg/dL)	Treatment with steroids did not affect renal recovery at 6 months (67.1% vs. 50.0%, *P* = 0.154)

*eGFR*, estimated glomerular filtration rate, *sCr*, serum creatinine, *ns* not significant

^a^Idiopathic/others (48%), drug induced (25%), tuberculosis (13%), sarcoidosis (9%), tubulointerstitial nephritis and uveitis (3%), and Sjögren syndrome (2%)

^b^Drug induced (67%), idiopathic (20%), tubulointerstitial nephritis and uveitis (8%), and sarcoidosis (4%)

^c^Drug induced, idiopathic, tubulointerstitial nephritis and uveitis, and others; but proportions were not described

The pathophysiology of ATIN is likely to be associated with hypersensitivity and immune reactions [9, 10], and steroid use is believed to have favorable effects on such mechanisms [24]. However, the detailed mechanisms of ATIN are not clearly understood, and thus, a treatment option for ATIN is currently limited. In addition to steroids, there could be other treatment options such as immunosuppressive agents, which are used to reduce the duration of steroid use or to treat steroid-refractory cases. Mycophenolate mofetil was introduced after steroids in patients with ATIN [25]. Additionally, other agents, including azathioprine and cyclosporine, were also prescribed in some patients [17]. Recently, rituximab was administered in immunoglobulin G4–related ATIN and reported to be effective in a case report [26]. However, these agents have not been proven as superior to steroids and are not easily applicable to current clinical practice.

Because patients with ATIN occasionally showed peripheral eosinophilia and tissue eosinophilic infiltration as well as extra-renal manifestations (e.g., skin rash), the pathophysiology of ATIN had been considered to be related with hypersensitivity reaction or Th2 cytokines [9, 10]. The present study showed that inflammatory cytokines and chemokines were elevated in patients with ATIN compared with healthy individuals. Most of patients with ATIN have a Th1 response with elevated levels of TNF-α, monocyte chemo-attractant protein-1, IL-6, and IL-8 in both urine and plasma. The pattern of cytokine levels elevated in ATIN was similar to that in renal ischemia-reperfusion model, although some cytokines (e.g., INF-γ) were not evident in patient samples. Furthermore, levels of IL-10 and IL-33, as representative Th2 cytokines, were not elevated in our patients. The findings suggest that pathogenesis of ATIN may be different from that of typical asthma or allergic rhinitis, which are known as Th2- or hypersensitivity-related disease. Intriguingly, IL-12p70 levels, related to NK cells activity [27], were elevated in urine, but not plasma, of patients.

The underlying mechanisms could not be determined by the present observations alone, but the cytokine assay may provide a clue for investigating another potential treatment agent. Many cytokine-targeting agents are currently used in clinical practice such as TNF-α inhibitor [28], and novel agents are in course of preparation to various diseases such as IL-17Rα inhibitor [29]. This cytokine analysis may be helpful in future application of treatment on ATIN.

Although our data are informative, there are some limitations. The present study was an observational study and not a randomized, controlled trial on steroid use. Accordingly, trials under control of baseline are required for a definite conclusion. The definition of renal recovery in the study focused on azotemia, and improvement in proteinuria could not be evaluated due to lack of follow-up data on urinalysis. Additionally, there were many unknown etiologies in this study, and this precluded from performing subgroup analysis by etiology. Cytokine levels after kidney biopsies were not examined, which may provide more insights on pathophysiologic significance.

## Conclusion

Steroid use has minimal effect on the long-term course of ATIN. Cytokine and chemokine results may help to identify the pathophysiology of ATIN and to develop new targeted therapies for ATIN in a future study. A further study on the mechanism and its translation are required to develop novel agents against ATIN and to finally improve patients’ outcomes.

## Additional file

Additional file 1: **Table S1.** Multivariable-adjusted logistic and Cox regression models of renal outcomes. (DOCX 22 kb)
