# Supplementary material for: Effect of steroids and relevant cytokine analysis in acute tubulointerstitial nephritis
Source: BMC Nephrol. 2019 Mar 12;20:88. doi: 10.1186/s12882-019-1277-2 (PMC6417076; doi:10.1186/s12882-019-1277-2)
Supplement: Supplementary file 1 — Table S1. Multivariable-adjusted logistic and Cox regression models of renal outcomes. (DOCX 22 kb) [file 12882_2019_1277_MOESM1_ESM.docx]

Table S1. Multivariable-adjusted logistic and Cox regression models of renal outcomes

| Variable | Renal recovery at 6months | | Renal recovery at the last follow-up | | End-stage renal disease | | All-cause mortality | |
| --- | --- | --- | --- | --- | --- | --- | --- | --- |
|  | OR (95% CI) | *P*-value | HR (95% CI) | *P*-value | HR (95% CI) | *P*-value | HR (95% CI) | *P*-value |
| Age (per 1year) | 1.01 (0.970**–**1.041) | 0.793 | 0.99 (0.974**–**1.011) | 0.405 | 1.01 (0.971**–**1.055) | 0.571 | 1.16 (1.066**–**1.260) | 0.001 |
| Female (vs. male) | 1.53 (0.580**–**4.019) | 0.392 | 1.04 (0.618**–**1.738) | 0.891 | 1.13 (0.382**–**3.323) | 0.828 | 0.69 (0.162**–**2.915) | 0.611 |
| Body mass index (per 1kg/m²) | 1.14 (0.978**–**1.325) | 0.095 | 1.06 (0.988**–**1.139) | 0.105 | 0.73 (0.606**–**0.874) | 0.001 | 1.06 (0.897**–**1.240) | 0.520 |
| Comorbidities (vs. none) |  |  |  |  |  |  |  |  |
| Diabetes mellitus | 0.28 (0.099**–**0.777) | 0.015 | 0.82 (0.466**–**1.448) | 0.496 | 0.87 (0.254**–**3.002) | 0.829 | 2.82 (0.678**–**11.747) | 0.154 |
| Hypertension | 0.66 (0.215**–**2.017) | 0.464 | 0.78 (0.437**–**1.386) | 0.394 | 5.11 (1.078**–**24.253) | 0.040 | 0.31 (0.066**–**1.486) | 0.144 |
| Chronic kidney disease | 0.52 (0.107**–**2.488) | 0.409 | 0.45 (0.165**–**1.221) | 0.117 | 9.11 (2.705**–**30.688) | 0.000 | 0.86 (0.115**–**6.484) | 0.886 |
| Laboratory findings |  |  |  |  |  |  |  |  |
| sCr at biopsy (per 1mg/dL) | 1.20 (0.944**–**1.530) | 0.136 | 1.11 (0.971**–**1.267) | 0.128 | 1.21 (0.986**–**1.485) | 0.067 | 1.00 (0.738**–**1.363) | 0.985 |
| Uric acid (per 1mg/dL) | 0.96 (0.776**–**1.180) | 0.680 | 0.96 (0.871**–**1.063) | 0.447 | 0.87 (0.677**–**1.129) | 0.302 | 0.83 (0.602**–**1.146) | 0.259 |
| Albumin (per 1g/dL) | 1.11 (0.902**–**1.357) | 0.333 | 1.02 (0.952**–**1.091) | 0.579 | 0.45 (0.144**–**1.400) | 0.167 | 0.57 (0.163**–**1.956) | 0.368 |
| Proteinuria (per 1 score) | 1.31 (0.788**–**2.167) | 0.300 | 1.00 (0.751**–**1.329) | 0.994 | 1.55 (0.917**–**2.621) | 0.102 | 1.51 (0.773**–**2.956) | 0.228 |
| Hematuria (vs. none) | 0.83 (0.264**–**2.601) | 0.747 | 1.47 (0.820**–**2.622) | 0.197 | 0.87 (0.268**–**2.830) | 0.818 | 0.97 (0.144**–**6.559) | 0.977 |
| Renal pathology (per 1 score) |  |  |  |  |  |  |  |  |
| TA/IF | 0.89 (0.482**–**1.656) | 0.721 | 0.87 (0.626**–**1.199) | 0.387 | 1.53 (0.743**–**3.163) | 0.247 | 1.44 (0.588**–**3.500) | 0.428 |
| Leukocytes infiltration | 0.83 (0.411**–**1.680) | 0.607 | 0.96 (0.675**–**1.352) | 0.796 | 0.90 (0.448**–**1.790) | 0.754 | 0.29 (0.089**–**0.951) | 0.041 |
| Dialysis at biopsy (vs. none) | 1.95 (0.494**–**7.699) | 0.340 | 1.20 (0.568**–**2.540) | 0.632 | 1.51 (0.436**–**5.258) | 0.514 | 4.08 (0.847**–**19.648) | 0.080 |
| Steroid use (vs. none) | 1.05 (0.258**–**4.269) | 0.947 | 0.82 (0.401**–**1.674) | 0.583 | 0.76 (0.188**–**3.032) | 0.693 | 0.33 (0.049**–**2.174) | 0.248 |

OR, odds ratio; HR, hazard ratio; CI, confidence interval; sCr, serum creatinine; TA/IF, tubular atrophy and interstitial fibrosis.
